# Congenital Melanocytic Nevi with Primary Cerebral Melanoma: A Rarity

**Published:** 2013-11-03

**Authors:** Xin Chen, Jing ZhiMao, Hong YuZhuo, Guang Peng, Xin Wu, Yi Feng, Feng Wang

**Affiliations:** Department of Medical Oncology, Cancer Center, State Key Laboratory of Biotherapy,West China Hospital, West China Medical School, Sichuan University, No. 37, GuoXue Xiang, Chengdu 610041, Sichuan Province, China.

**Keywords:** Congenital melanocytic nevi, Intracranial melanoma, Primary melanoma

## Abstract

A16-year-old male was admitted in hospital because of frontal and temporal melanoma. There were congenital giant nevi on his back and head. Positron emission computed tomography revealed no extra-cranial primary lesion. He underwent surgery, whole-brain radiotherapy (WBRT), and chemotherapy; but he could not be saved and died 6 months after establishing diagnosis.

## INTRODUCTION

Congenital melanocytic nevi (CMN) are benign proliferation of cutaneous melanocytes and often become apparent within the first few weeks of birth. The incidence of CMN in infants is 0.2-2.1% with a slight preponderance in female (M:F 1:1.17).[1-4] CMN is commonly regarded as a risk factor for malignant melanoma. Additionally, lesions on the trunk have higher risk of developing cutaneous melanomas.[5] The major medical concern with giant CMN is high risk of developing cutaneous melanoma, leptomeningeal melanoma, and neurocutaneous melanocytosis. But such a primary intracranial development is exceedingly rare. We report a case of primary intracranial melanoma with CMN.

## CASE REPORT

A 16-years-old male presented with impaired vision for 10 days and headache for 5 days prior to admission. He also suffered occasional mild abdominal pain and vomiting. No neurological symptoms or unusual behavior were observed. He was born with congenital giant nevi on trunk and head. Physical examination revealed many brownish-black nevi on the trunk, including a giant one covering nearly the entire back (35cm x 22cm). Another cephalic patch (5cm x 4cm) was situated on the right temporal region. The CMN was smooth, elevated and hairless. A computed tomography (CT) scan of the head showed a hyperdense mass surrounded by edema and obvious bleeding visualized in the right insular lobe. The size of the lesion was approximately 5.1cm x 4.3cm. Right cerebral ventricle became narrow because of compression. Magnetic Resonance Imaging (MRI) revealed a lesion which was hypointense on T2-weighted imaging and hyperintense on T1-weighted imaging in the right insular lobe. The MRI imaging showed obviously inhomogeneous enhancement and edema after gadolinium contrast (Fig. 1a, 1b).

**Figure:**
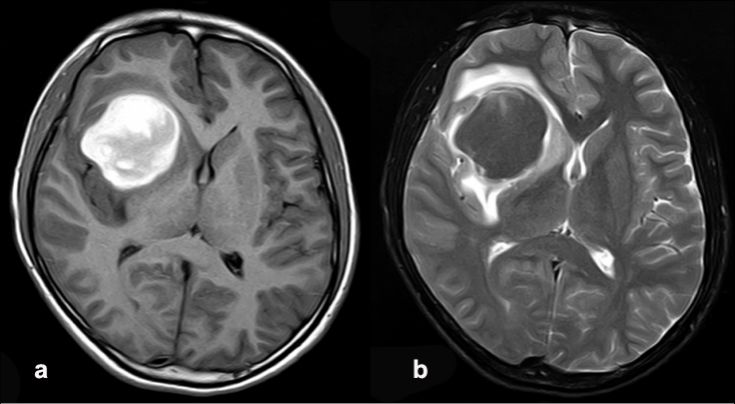
Figure 1:(a) Post-contrast T1-weighted image demonstrating heterogeneous enhancement of the lesion. (b) T2-weighted image demonstrating hypointense lesion in the right insular lobe with surrounding edema and hematoma.

At surgery, the cortical surface of the brain was normal. Pigmentation and hematoma were found on the frontotemporal sulcus during the operation. They were totally resected. The other pigmented mass was found to surround the frontal-side branch of the middle cerebral artery, which was 1.0 cm in diameter. As they were connected with the artery closely, a partial excision was done. Post-surgically pathological diagnosis of melanoma was made (Fig. 2). Immunohistochemical examination showed S-100(+), HMB 45(+),EMA(-),GFAP(-). In order to find possible origin of the lesion at other sites, abdominal ultrasound, radiographic examination including chest X-ray and whole body Positron Emission Computed Tomography (PET-CT) were done, but no pathology could be detected. He received WBRT and then chemotherapy containing cisplatin and dacarbazine after operation. The radiation dose was 59.4 Gy given in 27 fractions of 2.2 Gy each, with 5 fractions administered per week, starting after the diagnosis but before the first chemotherapy cycle. Unfortunately, the disease continued to progress in the follow up period. The patient died of multi organ failure 6 months after diagnosis of melanoma.

**Figure:**
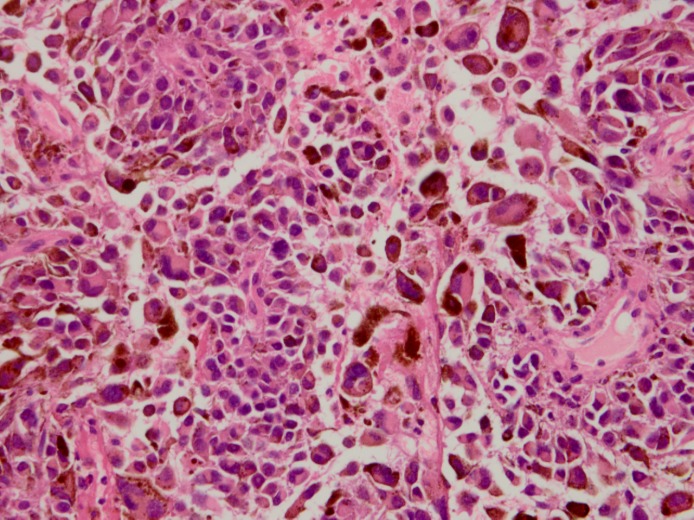
Figure 2:Photomicrographs of the tumor showing polygon, round type, spindle, singular cells. Tumor cells have large nuclei with inconstant big nucleoli. Mitotic figures are visible (x400).

## DISCUSSION

Malignant melanoma is the third most common tumor that metastasize to brain.[6] The primary malignant melanoma found in central nervous system accounts for approximately 1% of all the cases of melanomas.[7] They are usually found as leptomeningeal melanoma, and primary cerebellopontine angle and sellar melanocytic tumors are also reported.[8-10] As primary intraparenchymal melanoma are rare, a metastatic one was first to be considered. In order to find the “possible” extracranial site of origin, systemic evaluation was done in our patient with CT scan, MRI and PET-CT; however, no extracranial primary was demonstrated. Thus, based on the medical history of CMN, as well as a series of imaging investigations, we tend to conclude that the patient had a primitive melanocytic tumor.

Literature search showed 95% mortality in case of metastasis of melanoma to brain. Despite treatment, the median survival is less than a year[6] .The clinical outcome of patients with primary CNS melanoma is reported to be better than that of patients with metastatic disease.[11] Our patient had a poor prognosis. It has let us ponder if a complete excision would have been possible, followed by WBRT and chemotherapy, the outcome could be different. But in this case, the mass was surrounding the major vessels, thus limiting us to a partial excision.

## Footnotes

**Source of Support:** Nil

**Conflict of Interest:** None declared

